# Supplementary material for: Intrinsic catalytic properties of histone H3 lysine-9 methyltransferases preserve monomethylation levels under low S-adenosylmethionine
Source: J Biol Chem. 2023 Jun 17;299(7):104938. doi: 10.1016/j.jbc.2023.104938 (PMC10404681; doi:10.1016/j.jbc.2023.104938)
Supplement: Supporting Figures S1–S3 [file mmc1.pdf]

Figure S1: Comparison of HMT $K_{M,\ peptide}$ values with nuclear H3K9 proteoform availability.

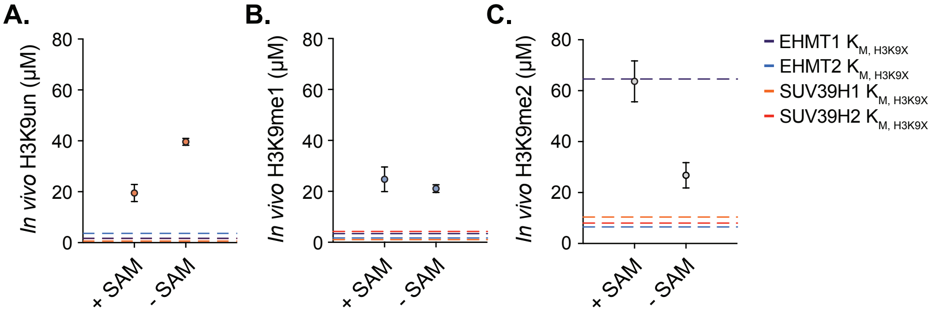

Figure S1: Comparison of HMT $K_{M,\ H3K9X}$ values with nuclear H3K9 proteoform availability. Nuclear (*A*) H3K9 unmodified (H3K9un), (*B*) monomethylated (H3K9me1), and (*C*) dimethylated (H3K9me2) residue concentrations under SAM-replete and -deplete conditions in HCT116 colorectal cancer cells (See Table 3). H3K9 peptide substrate K_M values generated from the kinetic analysis of EHMT1, EHMT2, SUV39H1, and SUV39H2 presented in Figure 3 are superimposed onto each graph to facilitate direct comparison of *in vivo* H3K9 proteoform availability with empirically determined *in vitro* H3K9 peptide proteoform K_M values.

Figure S2: Pseudo-single turnover kinetic analysis of EHMT1 on nucleosome substrates.

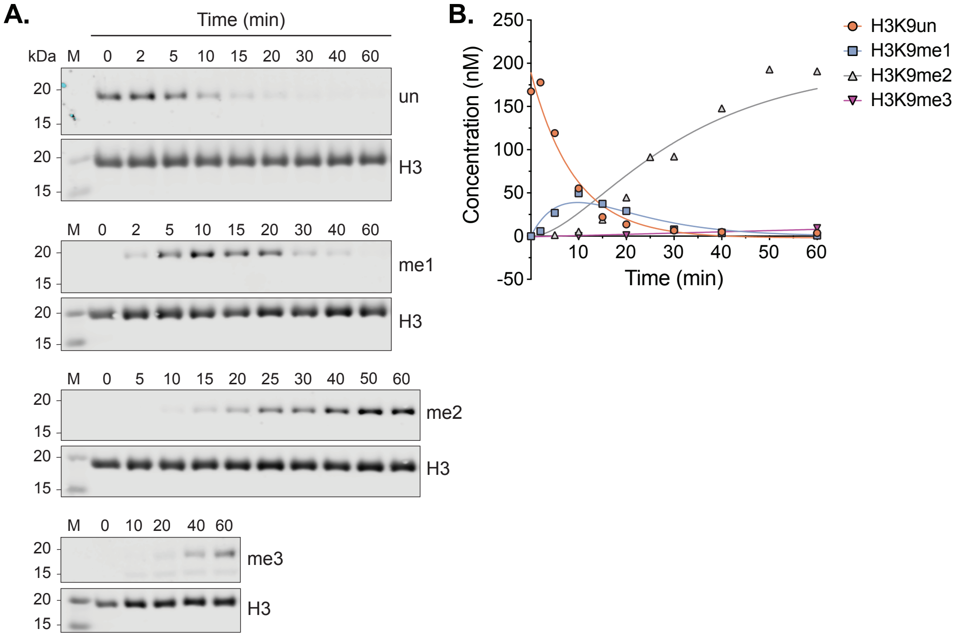

Figure S2: Pseudo-single turnover kinetic analysis of EHMT1 on nucleosome substrates. Quantification of EHMT1 (40 µM) pseudo-single turnover experiments with (*A-B*) unmodified, recombinant *H. sapiens* nucleosome substrates (100 nM). Western blot images used for the graphical quantifications are shown in panel (*A*). The H3K9 methylation state concentrations were determined by normalizing the methylated K9 signal to the total H3 signal. The k_{obs} values were derived from H3K9 methylation concentrations that were fit to consecutive reaction first-order rate equations (See Table 4).

Figure S3: HMT binding affinities for differentially methylated peptide substrates.

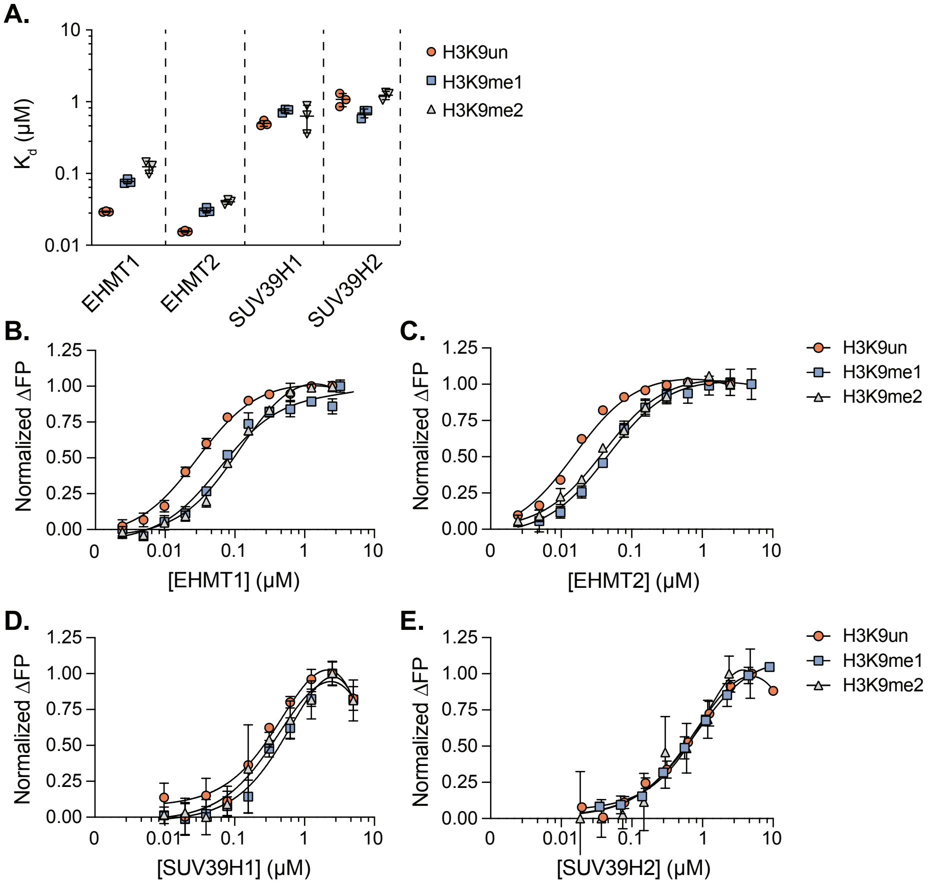

Figure S3. Comparison of HMT $H3_{(1\text{-}17)}$K9 proteoform dissociation constants. (*A*) Summary plot depicting K_d values derived from Fluorescence Polarization (FP) assays between EHMT1, EHMT2, SUV39H1, and SUV39H2 with 5-FAM labeled $H3_{(1\text{-}17)}$K9 unmodified (H3K9un), mono-methylated (H3K9me1), or di-methylated (H3K9me2) peptides. (*C-E*) Individual dose responses of EHMT1, EHMT2, SUV39H1, and SUV39H2 (0-10 μM) under fixed SAH concentrations (100 μM) and fixed $H3_{(1\text{-}17)}$ K9 peptide concentrations (varying based on peptide from 20-100 μM) which provided the summary values presented in panels (*A*). Data are presented as average ± s.d. from three technical replicates.
